# Sharing health research data – the role of funders in improving the impact

**DOI:** 10.12688/f1000research.16523.2

**Published:** 2018-12-24

**Authors:** Robert F. Terry, Katherine Littler, Piero L. Olliaro

**Affiliations:** 1 Research Policy, TDR - The Special Programme for Research and Training in Tropical Diseases, Geneva, 1211, Switzerland; 2 Policy Unit, Wellcome Trust, London, UK; 3 Implementation and Intervention Research, TDR - The Special Programme for Research and Training in Tropical Diseases, Geneva, 1211, Switzerland

**Keywords:** Health research, data sharing, public health emergencies, data standards, data infrastructure, pandemics, curation

## Abstract

Recent public health emergencies with outbreaks of influenza, Ebola and Zika revealed that the mechanisms for sharing research data are neither being used, or adequate for the purpose, particularly where data needs to be shared rapidly.

A review of research papers, including completed clinical trials related to priority pathogens, found only 31% (98 out of 319 published papers, excluding case studies) provided access to all the data underlying the paper - 65% of these papers give no information on how to find or access the data. Only two clinical trials out of 58 on interventions for WHO priority pathogens provided any link in their registry entry to the background data.

Interviews with researchers revealed a reluctance to share data included a lack of confidence in the utility of the data; an absence of academic-incentives for rapid dissemination that prevents subsequent publication and a disconnect between those who are collecting the data and those who wish to use it quickly.  The role of the funders of research needs to change to address this. Funders need to engage early with the researchers and related stakeholders to understand their concerns and work harder to define the more explicitly the benefits to all stakeholders.  Secondly, there needs to be a direct benefit to sharing data that is directly relevant to those people that collect and curate the data. Thirdly more work needs to be done to realise the intent of making data sharing resources more equitable, ethical and efficient.  Finally, a checklist of the issues that need to be addressed when designing new or revising existing data sharing resources should be created. This checklist would highlight the technical, cultural and ethical issues that need to be considered and point to examples of emerging good practice that can be used to address them.

## Introduction

Policies that require the sharing of health research data to improve public health have been promoted by international research funders for over a decade. However, when measured the quality and volume of health research data that has been shared, even when related to public health emergencies, remains low ^1, 2^. There are a number of ethical, legal and technical issues that act as impediments to sharing data but it seems this lack of progress is more a consequence of a cultural reluctance among researchers to ‘give up their data’ unless there are clear benefits returning to them. This reluctance is heightened among researchers in low resource settings who feel that the requirements to share data, from funders and journals, risk turning them into data exporters unless greater efforts are made to ensure a fairer distribution of benefits. In this paper the authors draw on their experience working for Wellcome Trust and TDR - the Special Programme for Research and Training in Tropical Diseases - of supporting data sharing initiatives and combine that with commissioned research to highlight the barriers to sharing research data and the role research funders might play to improve this situation.

## A decade of data sharing policies but little progress?

In January 2011, a group of research funding organizations published a joint statement on sharing health research data with the aim to harmonize their existing policies and promote the efficient use of those data to accelerate improvements in public health. The funders recognized that for data sharing to be most effective, a combination of technical and cultural issues need to be addressed. They framed this approach around three principles which required any data sharing mechanism they supported to be equitable, ethical and efficient ( See Wellcome Trust page on sharing research data). (See Box 1).

Box 1. **Sharing research data to improve public health the principles in the full joint statement by funders of health research (2010)**

**Equitable**: any approach to the sharing of data should recognise and balance the needs of researchers who generate and use data, other analysts who might want to reuse those data and the communities and funders who expect health benefits to arise from research.

**Ethical**: all data sharing should protect the privacy of individuals and the dignity of communities, while simultaneously respecting the imperative to improve public health through the most productive use of data.

**Efficient**: any approach to data sharing should improve the quality and value of research and increase its contribution to improving public health. Approaches should be proportionate and build on existing practice and reduce unnecessary duplication and competition.

Progress on encouraging the sharing of research data has been made over the subsequent decade and it is now common for research grants and journals to require the data underlying a paper or clinical trial to be shared (see PLOS editorial and publishing policies, AllTrials, and NIH data sharing policy.) However, recent public health emergencies with outbreaks of influenza, Ebola and Zika have brought into sharp focus the realization that the mechanisms for sharing data are neither being used or adequate for the purpose, particularly where data needs to be shared rapidly ^3– 5^.

In addition, researchers working in low- and middle-income countries highlight an inequity created by the disadvantage as they see it by the blanket requirements to share their data. Their concern is that sharing their data too soon, or without any restrictions will lead to their data being analysed by others with greater capacity, and no benefit will return to the researchers themselves or the populations they work with. In effect they become data exporters rather than partners. So while there is a lot of emphasis placed on data being Findable, Accessible, Inter-operable and Reusable, known as the FAIR approach, many researchers in developing countries fear the reality for them will be far from fair ^6– 8^.

## The findings of two surveys and a workshop

To explore this further, Wellcome and TDR commissioned two surveys to review the governance arrangements and standards within existing data sharing resources. The findings of those studies informed a workshop held in October 2017 with a set of stakeholders representing researchers and funding organizations with experience of sharing and using shared data.

All the reports, methods and supporting data files from these commissioned studies are published as open access under a Creative Commons licence and in free-to-access repositories. Readers are strongly encouraged to read that material as the primary source of reference ^1, 2, 9, 10^.

The first survey – Data Sharing in Public Health Emergencies - focussed on data sharing in public health emergencies concerned with the pathogens named by the World Health Organization as of priority concern because of their epidemic or pandemic potential (see WHO list of Blueprint priority diseases). A review of all academic papers published since 2003 referencing these diseases was undertaken and attempts were then made to access the data underlying those publications via the web and through a direct survey of the corresponding authors. Interviews were undertaken with a range of people either conducting or supporting research in these areas and this was supplemented with a review of institutional policies, discussion documents and academic commentaries about standards and norms in data sharing. The detailed search workflow, a spreadsheet recording the results, and a bibliography of all papers deemed in scope are published ^1, 2^. The year 2003 was chosen as the start date for the review as this was the year of the last major SARS (Severe Acute Respiratory Syndrome) outbreak. The World Health Organization held a Global Meeting on the Epidemiology of SARS and the resulting consensus on what data needs to be shared and how is seen as contributing to the successful control of that public health emergency (see Consensus document on the epidemiology of severe acute respiratory syndrome (SARS)), 

The second survey - Development of International Standards for Online Repositories - was designed to identify which technical ‘standards’ were being used in data sharing infrastructure relating to the neglected diseases. Standards were identified following a review of publicly accessible information (via the web or publication) relating to three main areas each with a set of elements describing the standards under those areas. For a full description of the methods and results readers are referred to the final published report. ^9^.

A third report combined the findings of these two surveys referenced above and was used to shape thinking at a workshop held in Antwerp, Belgium in October 2017. The workshop brought together 26 experts representing agencies that included those that provide data sharing resources for diseases prevalent in low and middle income countries. ^10^.

## Summary of the findings

Sharing health research data currently remains the exception rather than the norm. The review of research papers, including completed clinical trials related to priority pathogens, found only 31% (98 out of 319 published papers, excluding case studies) provided access to all the data underlying the paper. While a few authors will provide the data on request, 65% of the papers (207 of 319) give no information on how to find or access the data. And the review of clinical trial registries, for trials on interventions for priority pathogens, reported an even worse picture. Only two trials out of 58 provided any link in their registry entry to the background data ^1, 2^.

Interviews with researchers revealed the reasons for a reluctance to share data included a lack of confidence in the utility of the data and therefore unwillingness to invest resources to prepare it to be shared; absence of academic-incentives for rapid dissemination that prevents subsequent publication (as opposed to the public health need) and a disconnect between those who are collecting the data and those who wish to use it quickly. A similar scepticism about how data might be used or misused, the potential harms to patients and the risks to the researcher sharing data that might reveal errors in their work, have been reported elsewhere ^8, 11^.

Table 1 summarises the survey findings that identified which standards are used to share research data for neglected diseases and what those standards cover with respect to data curation, governance, security and longevity. Whilst there is clearly no universal or single standard to cover all the three areas and the elements under them, technical guidance is available across all the areas when those standards are combined. The standards created by the Clinical Data Interchange Standards Consortium (CDISC) were included as the United States Food and Drug Administration (FDA) has required the use CDISC standards in a clinical trial data submission since 2017. Hence these are widely used in industry and CDISC is fast becoming the *de facto* standard for data labelling and meta data.

**Table 1.** Elements of interest addressed by data repository standards used in sharing data on the neglected diseases (adapted from Castillon *et al.* ^9^).

AREAS	ELEMENTS	TRAC	ISO 16363	WHO	ICSU	H3Africa	CDISC
**DATA** **CURATION**	**Metadata**	+	+	-	-	+	+
	**Discoverability**	-	+	-	+	+	-
	**Data Standardization**	-	-	-	-	-	+
	**Data Verification and Quality** **Assurance Procedures**	+	-	+	+	+	-
**SECURITY AND LONGEVITY**	**Encryption, Access Control** **& Other Security Measures**	+	+	-	+	+	-
	**Storage**	+	-	-	+	+	-
	**Data Backup**	+	+	+	-	-	-
	**Data Migration**	+	+	-	-	-	-
	**Sustainability of Funding** **(fee, free-to-access etc.)**	+	+	+	+	-	-
	**Data Preservation**	-	+	-	+	-	-
	**Succession Plan**	+	+	+	-	-	-
**GOVERNANCE**	**Legal Status**	+	+	+	-	-	-
	**Access and Terms of use**	+	+	+	+	+	-
	**Benefit sharing, Intellectual** **Property Issues**	+	+	-	-	+	-
	**Audit Procedures**	+	+	+	-	-	-

+ As stated in publicly available information - Information was not mentioned in the publicly available information. TRAC (Trustworthy Repositories Audit & Certification), ISO 16363 (International Standards for Clinical Trial Registries -Space data and information transfer systems, Audit and certification of trustworthy digital repositories), WHO (World Health Organization), ICSU (International Council of Scientific Unions World Data System), H3Africa (The Human Heredity and Health in Africa Initiative), CDISC (Clinical Data Interchange Standards Consortium).

## Conclusion: what are the next steps for the role for funders in support of data sharing

Many health research funders have a generic policy requiring research data to be shared in a manner that maximises health and societal benefit. While some biomedical areas, like genomics, have forged ahead in maximizing data sharing, across health research more generally there is very low compliance with these policies. In part this might reflect the limited guidance and additional resources offered by the same funders in supporting their researchers to understand and undertake data sharing to implement and monitor these policies in practice.

It appears the main barrier to sharing is not technical but cultural, with researchers remaining sceptical about the benefits to them of sharing data. For researchers in low-resource setting data sharing can even be seen as a threat that their data will be exported and exploited by others with little benefit returning to them.

Therefore, research funders should take stock and revise data sharing policies to provide incentive structures for researchers. One clear first step would be to engage early with the researchers and related stakeholders to understand their concerns and work harder to define the benefit of sharing beyond a general sense that sharing data is in the public interest. There are very few evidence-based case studies that describe clearly the public health benefit that was achieved following the sharing of research data. Funders should ensure better monitoring of the implementation of their policies and where such evidence exists that shared data added value this should be documented and disseminated. The overall purpose of sharing the data needs to be clear and ideally developed with input from data suppliers, secondary data users, potential end-users and beneficiaries, and if possible with input from the participants that are the source of those data. Concerns regarding privacy versus the secondary use of the data need to be explored and mechanisms put in place to balance the public benefit against potential risks to privacy and confidentiality.

Secondly, there needs to be a direct benefit to sharing data that is directly relevant to those people that collect and curate the data. For example academics require citation of their work, including a data set. The generation of data and its subsequent citation for reuse needs to be integrated into research assessment – an idea captured in the Declaration on Research Assessment (see San Francisco Declaration on Research Assessment). Support for new mechanisms to publish data and papers rapidly during an emergency with peer review happening post-publication should serve both the need to share data and credit researchers.

So if the purpose of the data sharing mechanism is clear and all stakeholders buy into that purpose and if they feel their inputs will be recognised in research assessment together this will create a strong incentive to share. This was certainly our experience when working with Schistosomiasis researchers ^8^.

Thirdly, whilst there are a myriad of data standards to work with to meet the general principles of making data FAIR, more work needs to be done to realise the intent of making data sharing resources more equitable, ethical and efficient. As evident in the surveys summarized here good practice is starting to emerge so what is needed is better ways to share that practice. Funders need to work with the researchers and their networks to support the technical work required to develop standards that enable inter-operability. Alongside support for technical standards there needs to be sustainable support for the infrastructure necessary to host those data with the appropriate governance mechanisms to ensure the efficient, ethical and equitable access outlined in the joint statement by funders of health research (2010) referenced above.

One contributory role for funders would be to systematically collect the data management plans that they have requested as part of funding grants and make them publicly accessible. In line with good practice these should be standardized where possible and ideally have clear, machine-readable metadata. An online resource that brings together the reference material and policies that are exemplars of good practice in each of the categories that cover governance, data curation, security and longevity would provide the basis for a framework to guide the future development of new sharing resources.

Finally, a checklist of the issues that need to be addressed when designing new or revising existing data sharing resources should be created. In addition to defining the purpose of data sharing this would highlight the the political, technical, cultural, legal and ethical issues that need to be considered and point to examples of emerging good practice that can be used to address them. The authors are working on this next stage and hope that with this type of planning and support in place the data sharing long desired by research funders will start to become the norm. 

## Data availability

All data associated with this article are referenced and available as open access under a Creative Commons licence (CC BY).
