# Supplementary material for: Sharing health research data – the role of funders in improving the impact
Source: F1000Res. 2018 Dec 24;7:1641. Originally published 2018 Oct 15. [Version 2] doi: 10.12688/f1000research.16523.2 (PMC6317492; doi:10.12688/f1000research.16523.2)
Supplement: Peer review history

# Peer review: Referee response for version 2

**DOI:** 10.5256/f1000research.19270.r42207

**Published:** 2019-01-07

**Authors:** Amanda Blatch-Jones

**Affiliations:** 1 National Institute for Health Research Evaluation, Trials and Studies Coordinating Centre (NETSCC), University of Southampton, Southampton, UK

I have reviewed the revised version of the paper and the authors have responded and addressed my comments

I have read this submission. I believe that I have an appropriate level of expertise to confirm that it is of an acceptable scientific standard.

# Peer review: Referee response for version 2

**DOI:** 10.5256/f1000research.19270.r42205

**Published:** 2019-01-02

**Authors:** Phaik Yeong Cheah

**Affiliations:** 1 Mahidol Oxford Tropical Medicine Research Unit, Faculty of Tropical Medicine, Mahidol University, Bangkok, Thailand

Thank you for the updated paper. I have no further comments.

I have read this submission. I believe that I have an appropriate level of expertise to confirm that it is of an acceptable scientific standard.

# Peer review: Referee response for version 2

**DOI:** 10.5256/f1000research.19270.r42206

**Published:** 2018-12-28

**Authors:** Keith C. Norris

**Affiliations:** 1 Divisions of Health Services Research and Nephrology and Hypertension, Department of Medicine, David Geffen School of Medicine, University of California, Los Angeles, Los Angeles, CA, USA

The authors have addressed all of my concerns and I approve the revised manuscript.

I have read this submission. I believe that I have an appropriate level of expertise to confirm that it is of an acceptable scientific standard.

# Peer review: Referee response for version 1

**DOI:** 10.5256/f1000research.18059.r40330

**Published:** 2018-12-03

**Authors:** Keith C. Norris

**Affiliations:** 1 Divisions of Health Services Research and Nephrology and Hypertension, Department of Medicine, David Geffen School of Medicine, University of California, Los Angeles, Los Angeles, CA, USA

This is well thought through and timely opinion piece on the barriers to research data sharing, especially for issues of public health emergencies.

 The authors report on the findings from two related surveys and a workshop, and of note reported out of 319 published papers, excluding case studies, only 98 (31%) provided access to all the data underlying the paper.

 They also reported 65% of these papers gave no information on how to find or access the data (does the 65% refer to the 319 papers or the 98?).

 They provide several recommendations, but outside of the checklist the recommendations seem more aspirational and more about why one would want to or should share data. But as framed are not easily actionable.

 First - Funders need to engage early with the researchers and related stakeholders to understand their concerns and work harder to define the more explicitly the benefits to all stakeholders. 

 Second - there needs to be a direct benefit to sharing data that is directly relevant to those people that collect and curate the data.

 Third - more work needs to be done to realize the intent of making data sharing resources more equitable, ethical and efficient. 

 Finally - a checklist of the issues that need to be addressed when designing new or revising existing data sharing resources should be created. This checklist would highlight the technical, cultural and ethical issues that need to be considered and point to examples of emerging good practice that can be used to address them.

 The discussion needs to capture more of the following:

 There are legitimate concerns about early or premature data sharing that are not discussed, especially focusing on low resource research entities and the power dynamics that leverage the early release of data to the public as an opportunity for resource intensive researchers to leverage the data not only for “public good” but for their gain with no need for attribution to or sharing of downstream resources with the low resource entities that conducted the work. While many health research funders have a generic policy requiring research data to be shared in a manner that maximize health and societal benefit, these are often unfunded mandates, especially for research conducted in low resource countries or by low resource institutions.

 Releasing data in the absence of the context of much of the data can lead to significant misinterpretation of the data. Also, for certain data there needs to be time to clean and collapse the data to ensure participant privacy before it can be made publicly available. This can be a laborious process to do correctly and in many instances there may not be funding for this provided by the funder and low resource institutions may not have resources to conduct this unfunded work in an expeditious manner.

 For WHO pathogen work it seems funders might consider an a priori explicit plan of a cooperative agreement/partnership for early shared data for studies led by low resource “partners” doing the hands-on work and a more resource intensive partner (or funder) with capacity to do intense data analysis, with shared credit and equity in leveraging the data to get more funding for both partners. This can help to balance the power dynamics, and support early widespread data dissemination with the resource intensive partner (or funder) co-leading rapid production of publications and the creation of publicly available cleaned data for public benefit.

I have read this submission. I believe that I have an appropriate level of expertise to confirm that it is of an acceptable scientific standard, however I have significant reservations, as outlined above.

# Peer review: Referee response for version 1

**DOI:** 10.5256/f1000research.18059.r40329

**Published:** 2018-11-19

**Authors:** Amanda Blatch-Jones

**Affiliations:** 1 National Institute for Health Research Evaluation, Trials and Studies Coordinating Centre (NETSCC), University of Southampton, Southampton, UK

Sharing research data is important and the authors provide some valid points for consideration by funders and researchers. The article could do with some revisions in order to better flow and understand more about the survey and interviews. 

 The abstract doesn't state that two surveys and interviews were conducted and when - this would help bring paragraph 1 and 2 together. Also, the review of papers, was this a literature review/systematic review?

 The third paragraph could do with some revisions especially the first sentence. I wasn't sure what "Funders need to engage early with the researchers and related stakeholders to understand their concerns and work harder to define the more explicitly the benefits to all stakeholders." sentence means? What stakeholders?

 Introduction - improve what situation? I think this needs teasing out more in the first paragraph. Improving what - the quality of data to be shared, data sharing policies?

 What group of funders - who was involved? They framed this approach....Who framed?

 Highlighting that data collected specifically to answer a specific question in a trial for example, may not be sufficient to answer a different question, is important. Understanding why and how the data were collected, analysed and interpreted is important when reviewing secondary data. If this is misinterpreted, there are risks associated to the use of that data. 

 Findings of the two surveys and workshop - Who was involved in the survey, how many people involved and what was the response rate? Were funders involved in this? (For both of the surveys)

 Why was the review from 2003, given that data sharing para from 2011? As expected the results of the review are low, so are these numbers from recent research? What is the time period of those that made access to data? Did you see a change over time, those identified are from 2015 onwards for example. 

 Who participated in the workshop? Agencies from where? I think this would be important given the focus on low to middle income countries. 

 How many people interviewed? And who were they? Was this semi-structured/structured interviews, and how did you interpret the transcripts? Providing some context will help determine the value of the comments and confidence about the interpretation of the interviews. 

 Concluding section - Ethical issues may also need to be reviewed, are participants made aware at time of consent that the data collected for the specific study can later be used [secondary data] for other studies? Data sharing needs to be made aware to those who participate in the research. Transparency of use of data is needed at all levels, participants, researchers and funders. Funders have a role to implement and provide policies to ensure proper use of and sharing of data, and researchers have a role to ensure that they comply, acknowledge the use of the data and provide sufficient information about the data set and its intended purpose. 

 In terms of publicly accessible data management plans, is this the role of the funder and/or the researchers? If research protocols are 'meant' to be in the public domain then data sharing and statistical analysis plans should be as well? All funders approach these areas differently, so caveats around this may need to be mentioned. Possibly further consideration of these policies and how they could be standardised across funders, although consideration of early phase research to phase three studies may be required. 

 Is this applicable to industry and pharma? How does this relate to them considering the sensitivity of data?  How does this relate to AllTrials? (http://www.alltrials.net/) 

 How does data sharing fit into the wider agenda of transparency, accountability and credibility of research? Stating the larger viewpoint may strengthen the paper. 

I have read this submission. I believe that I have an appropriate level of expertise to confirm that it is of an acceptable scientific standard, however I have significant reservations, as outlined above.

# Peer review: Referee response for version 1

**DOI:** 10.5256/f1000research.18059.r39449

**Published:** 2018-10-25

**Authors:** Phaik Yeong Cheah

**Affiliations:** 1 Mahidol Oxford Tropical Medicine Research Unit, Faculty of Tropical Medicine, Mahidol University, Bangkok, Thailand

Here are my comments:

**Findings of two surveys and a workshop**

 First paragraph, first sentence - Who is “we”? The three authors of this paper?

 Second paragraph - The authors talked about a review of academic literature. Is this part of the survey or workshop? Perhaps this should be in the Introduction.

 Third paragraph – Suggest briefly stating the three main areas found in the survey.

 Fourth and fifth paragraph - Please tell us a little bit more about the third report and the workshop. 

**What does *this *tell us?**

 Does ***this*** refer to the findings of the two surveys and workshop?

 Paragraphs 2 and 3 – It is not clear what the objective of these paragraphs are. Do they summarise the barriers to data sharing? The reason I mentioned barriers is that in the Introduction, the authors said that this paper does two things – (1) to highlight the barriers to sharing research data and (2) the role research funders might play to improve this situation.

 If indeed they are barriers, are these barriers based on the two surveys and workshop or do they include research conducted by other groups? If the latter, I believe there are more barriers such as costs and ownership issues.

 Paragraph 2, first sentence. “Interviews with researchers”. Please provide reference(s). Did the authors conduct these interviews?

**What are the next steps: the role for funders in support of data sharing**

 Paragraph 1 – “…there is very low compliance with these policies”. How did the authors get this information? Please provide references if appropriate.

 Paragraph 2 - “It appears the main barrier to sharing is not technical but cultural,…” How did the authors get this information? Please provide references if appropriate.

 Paragraph 4 – “There needs to be direct benefit….” This is a general statement. What is the funders’ specific role?

 Suggest including a “Conclusions” section.

I have read this submission. I believe that I have an appropriate level of expertise to confirm that it is of an acceptable scientific standard, however I have significant reservations, as outlined above.
